# Clinical research on point-of-care lung ultrasound: misconceptions and limitations

**DOI:** 10.1186/s13089-024-00368-3

**Published:** 2024-05-10

**Authors:** Giovanni Volpicelli, Serena Rovida

**Affiliations:** 1 Department of Medical and Surgical Science, Magna Graecia University, Catanzaro, Italy; 2 Intensive Care Unit, St Georges University Hospital, London, UK; 3 Università degli Studi “Magna Graecia”, Azienda Universitario-Ospedaliera “Dulbecco”, Policlinico “Mater Domini” - Campus Universitario, Viale Europa, Germaneto, Catanzaro, 88100 Italia

## Abstract

Over the last 20 years, advances in point-of-care lung ultrasound (PoCLUS) have been consistent. The clinical application of PoCLUS has drastically changed the diagnosis of some respiratory conditions mainly in the acute setting. Despite these improvements, misconceptions regarding the current scientific evidence and errors in the direction given to the latest research are delaying the implementation of PoCLUS in the clinical field. The diagnostic power of PoCLUS is still under-evaluated in many settings and there is a generalized yet unjustified feeling that further evidence is needed before introducing PoCLUS as a standard of care. In the effort to build up further evidence by new studies, the role of randomized clinical trials is over-emphasized and gold standards used to investigate diagnostic accuracy of PoCLUS are sometimes not appropriate. Moreover, the sonographic patterns and techniques used to confirm the diagnoses not always are adapted to the patients’ clinical condition, which limit the scientific value of those clinical studies. Finally, there is a recurrent confusion in the role of PoCLUS scoring techniques, which should be only applied to quantify and monitor injury severity and not to diagnose lung diseases. Awareness of these misconceptions and errors could help the researchers when approaching new study projects on PoCLUS.

## Background

Point-of-care lung ultrasound (PoCLUS) is a highly impactful diagnostic tool that changed consistently the bedside approach to respiratory disorders over the last 15–20 years, especially in emergency and critical care settings. Within the overview of the most relevant diagnostic bedside novelties of these last years, the advent of PoCLUS can be considered as relevant as the introduction of the troponin essay for the evaluation of undifferentiated chest pain. Whilst troponin answers to a specific question and it is a relatively expensive test, PoCLUS covers a variety of different diagnostic applications at a very minimal cost. In 2012, the growing interest in PoCLUS and the rapid increase in literature led the scientific community to publish the international guidelines on the clinical use of lung ultrasound [1]. Lately, the document has become one of the most influential articles both in the field of general point-of-care ultrasound and critical care medicine [2, 3]. PoCLUS publications went from a little over 300 papers, which were analyzed for the first consensus document, to more than 2700 produced in the last 10 years [4]. In the era post Sars-CoV-2 pandemic the literature has almost doubled as PoCLUS has become the preferred diagnostic and monitoring imaging tool for CoViD-19 pneumonia.

Despite the scientific progress being unequivocable, there are few misconceptions currently holding back the implementation of PoCLUS. Unnecessary scientific evidence, unreasonable use of randomized controlled trials (RCT), inappropriate gold standards, imprecise lung patterns and techniques definition, confusion between diagnostic and scoring techniques, and lack of consideration of some intrinsic limitations are some of the aspects slowing down the expansion of PoCLUS in the daily clinical practice and reducing the efficacy of diagnostic studies. Thus, when approaching new studies and research protocols in PoCLUS applications, some crucial questions should be responded.

### Scientific evidence: do we need more?

Regardless of that fact that the utility and feasibility of PoCLUS has been largely proven in literature, its application in the daily clinical activity is still prevented by the common mindset that there is not yet enough evidence about its accuracy and superiority to conventional imaging [5–7]. Recent international guidelines on pneumothorax and pneumonia do not even mention PoCLUS among the variety of available diagnostic tools [8, 9]. As a general assumption, evidence will never be enough and the necessity to constantly better our scientific knowledge is an endless process. However, in this specific circumstance we should refine what the definition of “enough evidence for PoCLUS” means to push its clinical development even further. For instance, among the vast groups of PoCLUS application, one of the most debated remains the diagnosis of pneumothorax. Leading international guidelines do not promote the use of PoCLUS because considered not enough scientifically validated, and still encourage the use of conventional chest radiography (CXR) [7]. Nonetheless, there are many trials demonstrating that PoCLUS is highly accurate. When compared to CXR, PoCLUS has similar high specificity but remarkably superior sensitivity [10, 11]. Interestingly, in literature there are no trials investigating the accuracy of CXR for the diagnosis of pneumothorax. PoCLUS has also a well demonstrated diagnostic power for other conditions such as pulmonary edema, interstitial diseases, and pneumonia but remains underrated in the respective diagnostic guidelines. In the expert eyes, the conclusion outlined by international societies appears more like a prejudice rather than a scientific statement [12].

### Is the randomized clinical trial a correct approach?

A common mistake when it comes to research in PoCLUS is the need for a RCT to validate the diagnostic power of an imaging tool. RCTs are prospective studies that measure the effectiveness of a new intervention or treatment [13]. The strength of RCT relies on reducing bias and provide a tool that investigates relationships between an intervention and an outcome. RCTs are the most appropriate and powerful studies when new treatments must be compared with old ones or placebo [13]. Two or more treatments cannot be administered to the same patient, which justifies the recurrence to complex randomization. Hence, when a new diagnostic tool is non-invasive and can be used in combination with the conventional one, the need for expensive and labored RCTs is baseless [14]. In a previous RCT carried out to estimate the diagnostic accuracy of PoCLUS for heart failure, the study did not add any significant validation to what was previously demonstrated [15]. When comparing diagnostic accuracies of PoCLUS and CXR for acute decompensated heart failure it is possible to perform both exams in the same patient to assess non-inferiority or investigate superiority by also applying a gold standard imaging tool [16]. Studies demonstrating diagnostic non-inferiority give anyway an adequate contribution because of the intuitive advantages of PoCLUS in reducing radiation exposure, economic costs, and time [17].

### Is the diagnostic gold standard always adapted to the study aim?

PoCLUS relies on the interpretation of sonographic artifacts. It is possible to recognize and monitor different lung patterns due to loss of aeration with increase in the organ density. However, like all radiologic imaging methods, ultrasound cannot finalize diagnosis unless the detailed clinical context is taken into consideration [18, 19]. The gold standards adopted in PoCLUS research depend upon the aim of the study, whether we want to investigate the accuracy in the diagnosis of pulmonary patterns or intend to evaluate the ability to diagnose specific diseases. In the first case, the morphologic analysis of specific parenchymal patterns mandates the comparison with chest computed tomography (CT) as reference gold standard method [20, 21]. In the second case, when the aim is the diagnosis of a disease CT cannot be the gold standard, instead a clinical approach should be preferred [22]. An example of how sonographic patterns cover an umbrella of lung conditions is the diffuse interstitial B-lines pattern, which is commonly observed in cardiogenic edema, lesional edema of acute respiratory distress syndrome (ARDS), fibrosis, and viral infections. Comparison with CT will be useful to analyze correspondence of distribution and intensity of the interstitial lesions, but the clinical context will be essential for the final diagnosis when the study aim is to assess the role of PoCLUS in the diagnosis of specific diseases. Similarly, in studies whose aim is exploring the ability of PoCLUS in the diagnosis of lung consolidations, CT should be used as gold standard [20]. When the diagnosis of pneumonia is the endpoint, diagnostic confirmation needs an integrated clinical approach [23].

### Are similar but not identical patterns and techniques differentiated according to the study setting?

Definition of a lung pattern is crucial in PoCLUS research and varies according to patient’s condition and setting. An example is the definition of diffuse interstitial syndrome that significantly differs in between protocols. In the BLUE protocol, which applies to critically ill patients with acute severe respiratory failure, the interstitial syndrome is investigated in the anterior chest and defined as “anterior-predominant bilateral B-lines”, which confirms the diagnosis of cardiogenic pulmonary edema [24]. In settings where variable degrees of disease severity are evaluated, the ultrasound examination must extend to lateral chest regions and pulmonary congestion in cardiac failure is diagnosed by the presence of B-lines in “at least two antero-lateral positive scans per side” [25]. When fibrosis or interstitial pneumonia are the exam indications, analysis of B-lines must include the posterior chest [26, 27]. Although there are similarities, it is mandatory to specify which pattern definition and which technique we refer to in the research study. Another demonstration that patterns and techniques should be adapted to different settings is in studies on pneumothorax. PoCLUS diagnosis of pneumothorax banks on combination of different signs, including lung sliding, B-lines, lung pulse, and lung point, and consideration of the clinical context [28]. Without a correct combination PoCLUS accuracy would be strongly impaired. The absence of lung sliding is highly suspicious for pneumothorax in a specific clinical context, such as severe chest trauma [29]. In extreme emergency conditions the combination of absence of lung sliding, lung pulse, and B-lines is definitively diagnostic of pneumothorax. However, sliding is abolished in a variety of conditions other than pneumothorax, including ARDS, pneumonia, pleural adhesions, selective right mainstem intubation, and others. As a result, in a stable patient with absence of sliding it is mandatory to search for other sonographic signs to confirm the diagnosis. It is also important to remind that the presence of lung sliding allows to rule-out pneumothorax, but only in the chest region underneath the probe. Hence, we cannot make any assumption on the nearby regions until diligently scanned because, when the pneumothorax is relatively small, there are portions of the chest wall still showing a normal lung respiratory movement [30, 31].

### Are scoring techniques appropriately used for quantifying and monitoring and not for diagnosing?

PoCLUS can be used to diagnose lung disorders and can also quantify and monitor the severity of the lung condition over time. Diagnostic criteria are complex. They rely on basic lung patterns (interstitial syndrome, consolidations, respiratory sliding), but also on additional signs including air bronchograms, lung pulse, lung point, characteristics of the pleural line, distribution and intensity of the B-lines, shape, dimension, and margins of the consolidations, relationship with effusion, margins of re-aeration, and others. Techniques for quantification are based on few basic lung patterns, and exclusively validated to define the severity of the loss of aeration or the magnitude of pulmonary congestion. Criteria for scoring are relatively simple as well correlated with sophisticated volumetric tools and invasive determinations [21, 32, 33]. Methods for diagnosing and techniques for scoring are not interchangeable. Using scoring criteria to diagnose lung conditions or, vice versa, applying diagnostic methods to quantify severity of diseases are results in fallacies that could invalidate study results, as happened in recent literature [34–36].

### Are some intrinsic limitations of PoCLUS considered in the study protocol and corrected?

While PoCLUS being readily available, bedside, low cost, and radiation free tool to examine lung tissue, it has some limitations that need to be considered in research activities. Like any radiological method, PoCLUS has a high interoperator variability which depends almost exclusively upon the clinician skill. One of the strategies to reduce bias is running hands-on workshops prior to start a research study, aiming to bring all the operators at a standardized sonographic level in the shortest time [22]. Efficacy of PoCLUS can further drop in case the clinician performing the scan is not the same interpreting the images. Knowing how images where acquired helps to finalize the diagnosis and overcome misinterpretations. While CXR and CT have a conventional well standardized nomenclature, PoCLUS terminology is quite innovative. From the probe movement to the definition of lung patterns and signs, there is a whole new sono-dictionary for the clinician that still need to be assimilated and further standardized. However, inventing new terms and definitions does not contribute to clarity in the scientific community.

## Data Availability

No data are included in the manuscript.

## Abbreviations

PoCLUS: Point-of-Care Lung UltraSound
Sars-CoV-2: Severe Acute Respiratory Syndrome COronaVirus 2
CoViD-19: Corona Virus Disease 2019
RCT: Randomized Controlled Trial
CXR: Chest Radiography
CT: Computed Tomography
ARDS: Acute Respiratory Distress Syndrome
BLUE protocol: Basic Lung Ultrasound Examination protocol
